# Network and role analysis of autophagy in *Phytophthora sojae*

**DOI:** 10.1038/s41598-017-01988-7

**Published:** 2017-05-12

**Authors:** Linlin Chen, Xiong Zhang, Wen Wang, Xuejing Geng, Yan Shi, Risong Na, Daolong Dou, Honglian Li

**Affiliations:** 1 Department of Plant Pathology, Henan Agricultural University, Zhengzhou, 450002 China; 2 Department of Plant Pathology, Nanjing Agricultural University, Nanjing, 210095 China

## Abstract

Autophagy is an evolutionarily conserved mechanism in eukaryotes with roles in development and the virulence of plant fungal pathogens. However, few reports on autophagy in oomycete species have been published. Here, we identified 26 autophagy-related genes (*ATGs*) belonging to 20 different groups in *Phytophthora sojae* using a genome-wide survey, and core ATGs in oomycetes were used to construct a preliminary autophagy pathway model. Expression profile analysis revealed that these ATGs are broadly expressed and that the majority of them significantly increase during infection stages, suggesting a central role for autophagy in virulence. Autophagy in *P. sojae* was detected using a GFP-PsAtg8 fusion protein and the fluorescent dye MDC during rapamycin and starvation treatment. In addition, autophagy was significantly induced during sporangium formation and cyst germination. Silencing *PsAtg6a* in *P. sojae* significantly reduced sporulation and pathogenicity. Furthermore, a *PsAtg6a*-silenced strain showed haustorial formation defects. These results suggested that autophagy might play essential roles in both the development and infection mechanism of *P. sojae*.

## Introduction

Autophagy is a conserved cellular process in which cytoplasmic contents are degraded within a lysosome or vacuole, and the resulting macromolecular constituents are recycled^1, 2^. Cytoplasmic materials are degraded to produce amino acids and fatty acids during periods of autophagy^3^. Therefore, autophagy is essential for cell survival under various stress conditions, such as starvation^4^. Recent studies have revealed a wide variety of physiological roles for autophagy and its relevance to diseases. In many well-characterized plant pathogenic fungi, such as *Magnaporthe grisea*, *Fusarium graminearum* and *Ustilago maydis*, autophagy has been associated with development and virulence^5–9^.

During autophagy, cup-shaped, single-membrane-bound structures called isolation membranes appear and expand, resulting in cytosol and organelle sequestration^10^. Eventually, spherical, double-membrane-bound structures called autophagosomes are formed, which are delivered to lysosomes for fusion or vacuoles for degradation of contents^11^. This process requires the concerted actions of a distinctive set of proteins named ATG (autophagy-related). The core autophagy machinery can be divided into different subgroups: the Atg1 protein kinase complex (Atg1, Atg13 and Atg17–Atg31–Atg29 subcomplex) is essential for initiating induction^12^; the Atg14-containing phosphatidylinositol-3 kinase complex (beclin 1/Atg6, Vps34 and Vps15 with Atg14), which is essential for the recruitment of ATG proteins to the phagophore assembly site for vesicle nucleation; the Atg2-Atg18 complex and the Atg12-Atg5 protein conjugation system, which are involved in vesicle expansion and completion^13, 14^; and the Atg9-containing membrane protein recycling system^15^. *ATG* genes have been studied in many eukaryotic organisms, such as *Saccharomyces cerevisiae*, *Arabidopsis thaliana*, *Caenorhabditis elegans* and *Homo sapiens* but have received little attention in oomycete species^16–19^.

Oomycetes, classified in the kingdom Stramenopiles, contain many economically important eukaryotic plant pathogens, including *Phytophthora sojae* ^20^. Asexual sporangia (or zoosporangia) or zoospores play essential roles in both the initial infection and spread of *P. sojae* from host to host^21^. Zoospores are released from sporangia and guided by chemotaxis and electrotaxis towards host roots. Then, the zoospores germinate to form structures known as appressoria that breach the host epidermis. When the health of a colonized plant declines, sporangia and/or zoospores develop and move to another new host^22, 23^. In addition, *Phytophthora* species are hemibiotrophic pathogens, with a lifestyle that includes an abiotrophic phase, followed by a switch to necrotrophy^24, 25^. Although the physiological and genetic mechanisms of sporulation, spore germination, and hemibiotrophic disease cycles have been well-studied, little research has been performed on the role of autophagy in such processes.

In this study, 26 *ATG* homolog genes from the core autophagy machinery were identified, and a preliminary autophagy pathway model was posited based on core *ATGs* in oomycetes. *P. sojae* autophagy was induced with rapamycin and was also activated in *P. sojae* sporangia and germinating cysts. In addition, a gene orthologous to yeast *Atg6*, named *PsAtg6a*, was experimentally silenced in *P. sojae*, and the *PsAtg6a*-silenced transformants showed defects in sporangia production and virulence. These results will elucidate the effects of autophagy on the development and virulence of *P. sojae*.

## Results

### Identification of ATGs in *P*. *sojae*

**Figure 1:**
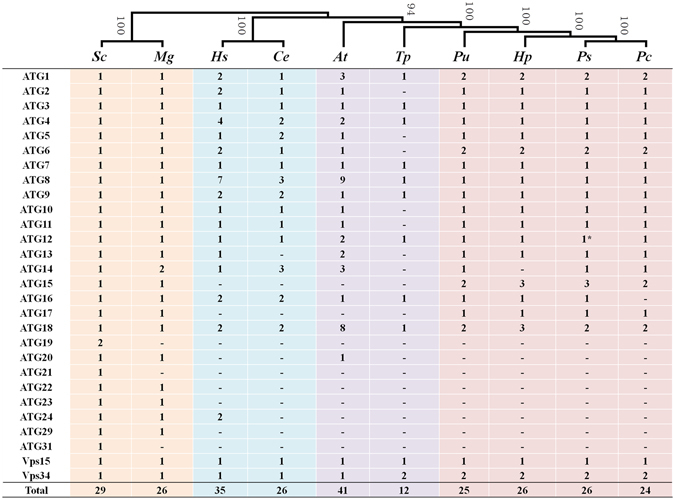
Distribution of autophagy-related (ATG) proteins in representative species. ATG proteins identified from *P*. *sojae* (*Ps*), *P*. *capsici* (*Pc*), *H*. *parasitica* (*Hp*), *Py*. *ultimum* (*Pu*), *T*. *pseudonana* (*Tp*), *A*. *thaliana* (*At*), *C*. *elegans* (*Ce*), *H*. *sapiens* (*Hs*), *S*. *cerevisiae* (*Sc*), and *M. grisea* (*Mg*) were compared. The distribution of putative ATGs appeared to be mostly restricted to oomycete species. Evolutionarily, the autophagy pathway of oomycetes appears more closely related to that of plants and animals than to that of fungi.

**Figure 2:**
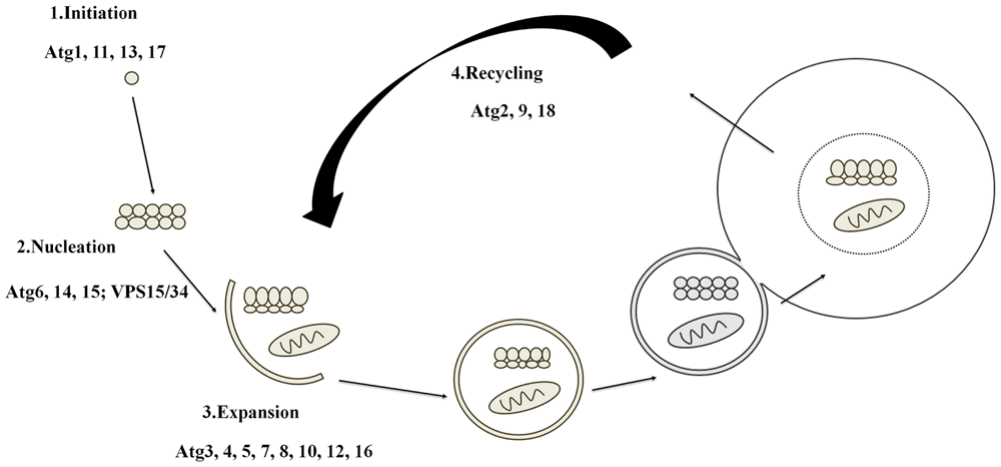
Schematic representation of autophagy in *P*. *sojae*. According to the distribution of ATGs in *P*. *sojae*, autophagy divided into 4 steps, as it is in *H*. *sapiens*: initiation of autophagy, vesicle nucleation at the PAS, vesicle expansion and recycling.

Several putative ATGs with conserved sequences and function have been identified in yeast, filamentous fungi, plant, and mammalian genomes^16, 17, 19, 26^. To identify ATGs in *P. sojae*, a tBlastn search was performed using different ATG sequences from *M. grisea*, *S. cerevisiae*, *A. thaliana*, *C. elegans* and *H. sapiens* ^27^. To further confirm putative ATG homologues in *P. sojae*, the deduced ATG protein sequences were analyzed in the Pfam database (http://pfam.sanger.ac.uk/) based on *S. cerevisiae* and *H. sapiens* ATGs. After removing redundant sequences, a total of 26 putative ATGs were identified in *P. sojae* (Fig. 1 and Supplementary Table S1). The information returned for each ATG is listed in Supplementary Table S1. As in other species, autophagy in *P. sojae* can be divided into 4 steps: initiation of autophagy, vesicle nucleation at the preautophagosomal structure (PAS), vesicle expansion, and recycling (Fig. 2). In addition, the ATG prediction pipeline, including building and updating species-specific hidden Markov models (HMMs), was used to predict ATGs in three other oomycetes (24 candidates in *Phytophthora capsici*, 25 candidates in *Pythium ultimum*, and 26 candidates in *Hyaloperonospora parasitica*) and one diatom (12 candidates in *Thalassiosira pseudonana*) (Fig. 1 and Supplementary Table S1). ATG proteins in these species are categorized based on their function in autophagic processes based on yeast and mammal studies^28, 29^. The putative ATG distribution was mostly restricted to oomycete species. Furthermore, oomycetes, unlike fungi (*M. grisea* and *S. cerevisiae*), lack Atg19, Atg20, Atg21, Atg22, Atg23, Atg24, Atg29 and Atg31 proteins, but Atg1, Atg6, Atg15, Atg18 and Vps34 are expanded, suggesting that the autophagy pathway of oomycete species is more closely related to animals and plants than to fungi.

**Figure 3:**
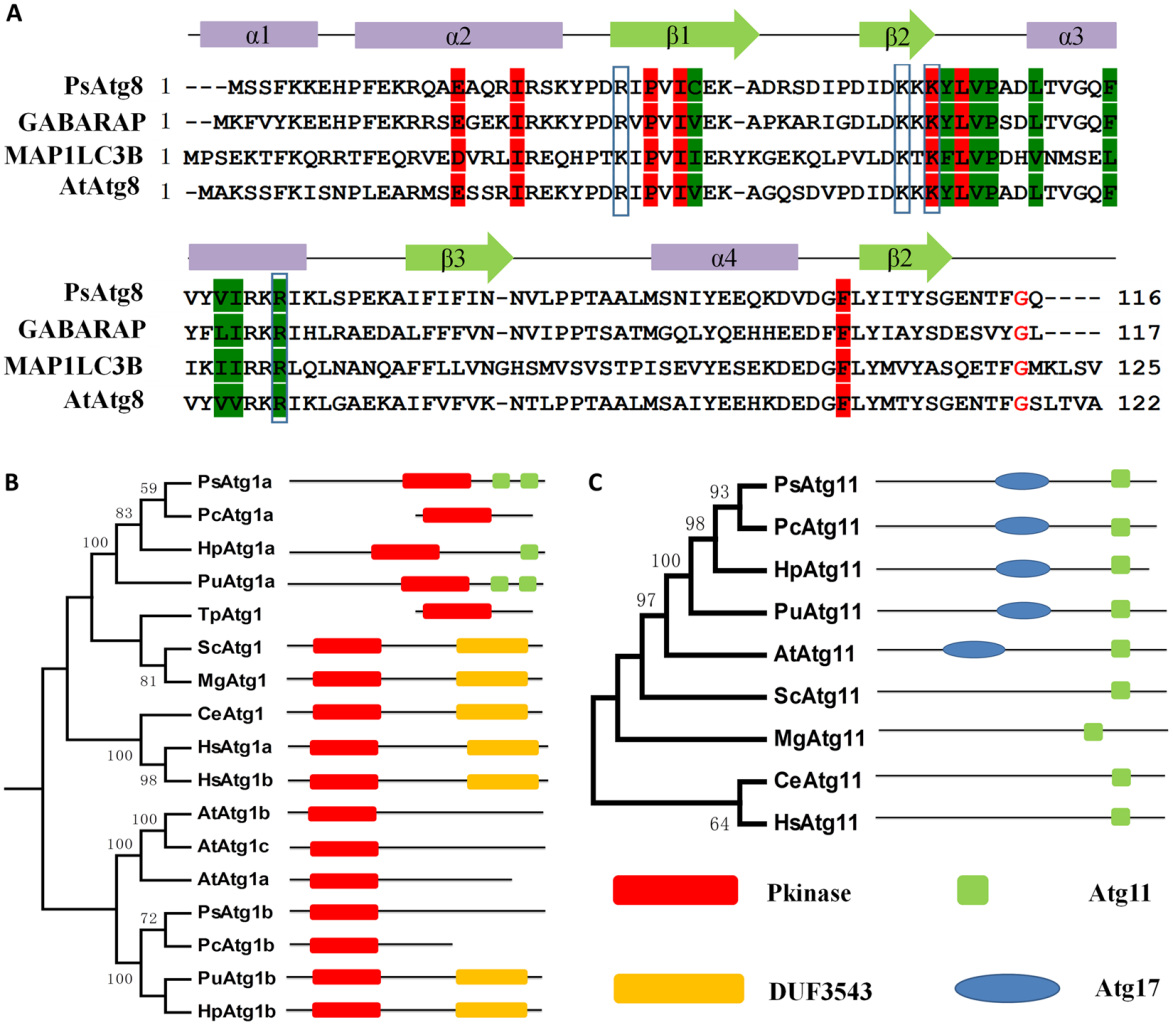
Phylogenetic relationship and domain structures of selected ATGs. (**A**) Sequence features of Atg8 proteins. Atg8 proteins from *H*. *sapiens* (GABARAP and MAP1LC3B), *A*. *thaliana* (AtAtg8) and *P*. *sojae* (PsAtg8) are shown. Atg8 proteins of different species are highly conserved across their entire length, and very similar to each other in most positions. (**B**) and (**C**) Phylogenetic relationship and domain structures of Atg1 and Atg11. The phylogenetic trees of Atg1 (**B**) and Atg11 (**C**) proteins identified from *P*. *sojae* (*Ps*), *P*. *capsici* (*Pc*), *H*. *parasitica* (*Hp*), *Py*. *ultimum* (*Pu*), *T*. *pseudonana* (*Tp*), *A*. *thaliana* (*At*), *C*. *elegans* (*Ce*), *H*. *sapiens* (*Hs*), *S*. *cerevisiae* (*Sc*), and *M. grisea* (*Mg*). Pkinase: kinase domain. Atg11: Atg11 domain. DUF3543: DUF3543 domain. Atg17: Atg17 domain.

In *P. sojae*, most ATGs seem to be highly conserved compared with different species (Supplementary Table S1). For example, putative Atg8 orthologs encode a similar amino acid sequence and domain structure to Atg8 proteins from other species^30^ (Fig. 3A). However, some ATGs, such as Atg1, Atg11 and Vps34, are specific to oomycetes (Supplementary Table S1 and Fig. 3). Atg1, the only kinase of the core autophagy machinery, is required for autophagosome formation and the Cvt pathway^31^. The oomycete protein Atg1b belongs to an ancient family that is conserved in almost all eukaryotic organisms, and the predicted protein shares a C-terminal Pkinase domain. However, an obvious difference from the above is that the DUF3543 domain in conserved PI3K is replaced by an Atg11 domain in Atg1a (Fig. 3B). In *S. cerevisiae*, Atg11 is the adaptor of the Cvt-specific receptor protein Atg19/Atg34^32^. However, an extra Atg17 domain aside from the Y-X-X-X-L/V/I-X-E-V/I-X-R-R-L sequences in oomycete Atg11 has been identified (Fig. 3C). In addition, a unique PI3K family that exists only in oomycetes has been reported in previous studies^33^. All of these data suggest that the core autophagy machinery is conserved in oomycetes with some differences in individual regulation.

### Expression profiles of *P*. *sojae ATGs*

**Figure 4:**
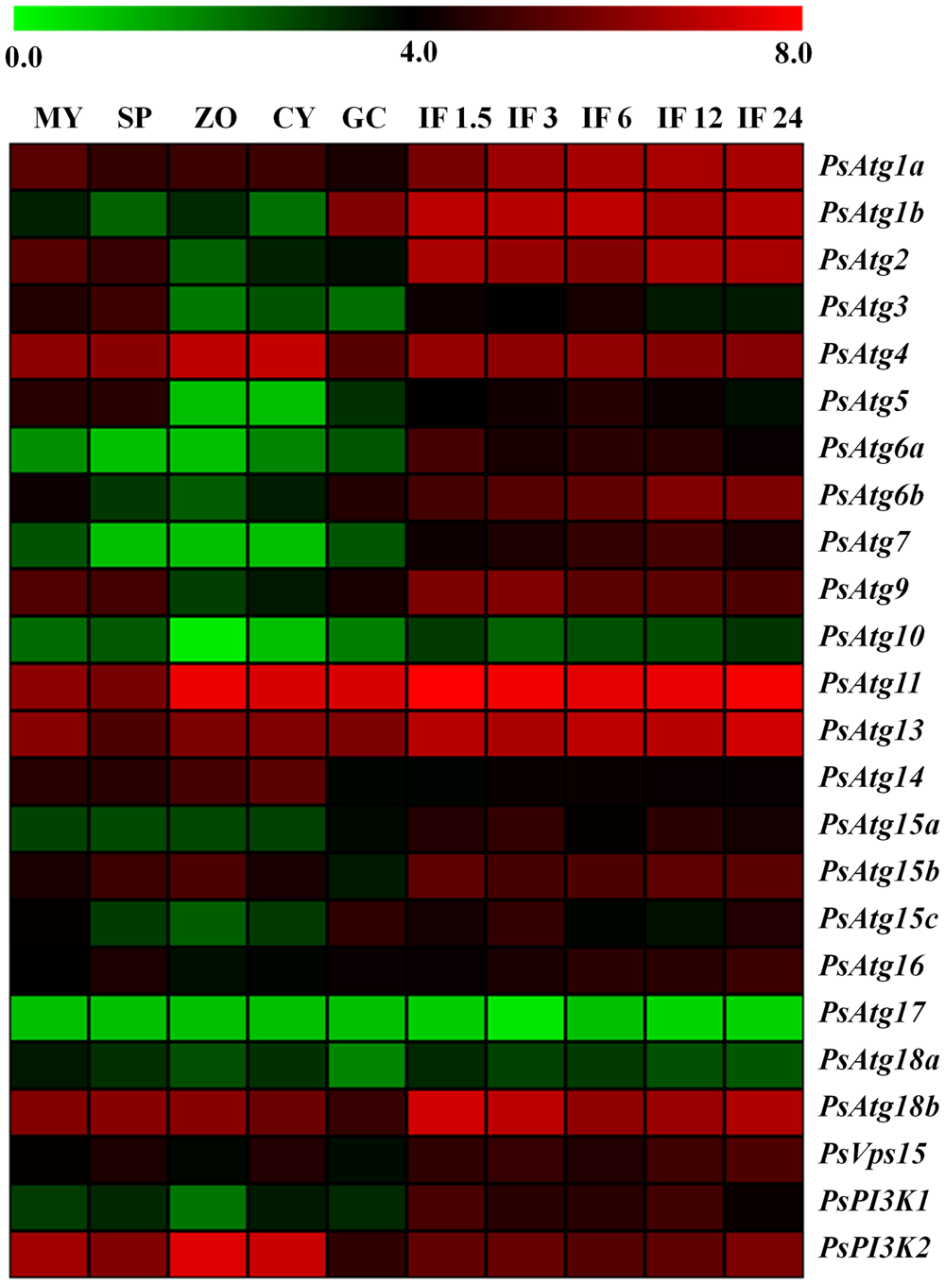
Heat map of expression profiles for *P. sojae ATGs*. Color bar represents the log2 expression values, ranging from green (0) to red (8). MY, mycelia; SP, zoosporangia; ZO, zoospores; CY, cysts; GC, germinated cysts; IF1.5 to IF24, indicates samples from 1.5, 3, 6, 12 and 24 h after infection of soybean leaves.

Increasing evidence suggests that autophagy is essential for the development and pathogenesis of many organisms^5^. To explore the potential role of ATGs in the development and virulence of *P. sojae*, the expression patterns of 24 *ATGs* (*PsAtg8* and *PsAtg12* were excluded) at 10 different stages were obtained from the published DGE transcriptional database^34^. Heat map analysis was performed based on the relative expression level of each *ATG* gene (Fig. 4). The majority of *ATGs*, including *PsAtg1a*, *PsAtg1b*, *PsAtg2*, *PsAtg6a*, *PsAtg6b*, *PsAtg7*, *PsAtg10*, *PsAtg11*, *PsAtg13*, *PsAtg15a*, *PsAtg15b*, *PsAtg16*, *PsAtg18b*, *PsVps15*, and *PsPI3K1*, show a relatively higher expression level (>2-fold expression change) during infection stages (2–14-fold; *P* ≤ 0.01) (Fig. 4), suggesting that these genes might play important virulence roles. In particular, *PsAtg1b* (9.0–12.7-fold) and *PsAtg6a* (7.0–14.0-fold) have a relatively high expression level of the assessed *ATGs*. Furthermore, the relative expression levels of 3 *ATG* genes (*PsAtg6a*, *PsAtg8* and *PsAtg9*) were validated by quantitative RT-PCR (Supplementary Fig. S1). The analysis showed that *PsAtg6a* was dramatically up-regulated during infection stages, whereas *PsAtg9* maintained a steady expression level; these results are comparable to transcriptome data. *PsAtg8*, which is required for autophagosome formation and is a reliable marker of autophagy induction and progression, visibly changes during infection (Supplementary Fig. S1). These data suggest that autophagy might function in *P. sojae* virulence.

### Autophagy induction by rapamycin in *P. sojae*

**Figure 5:**
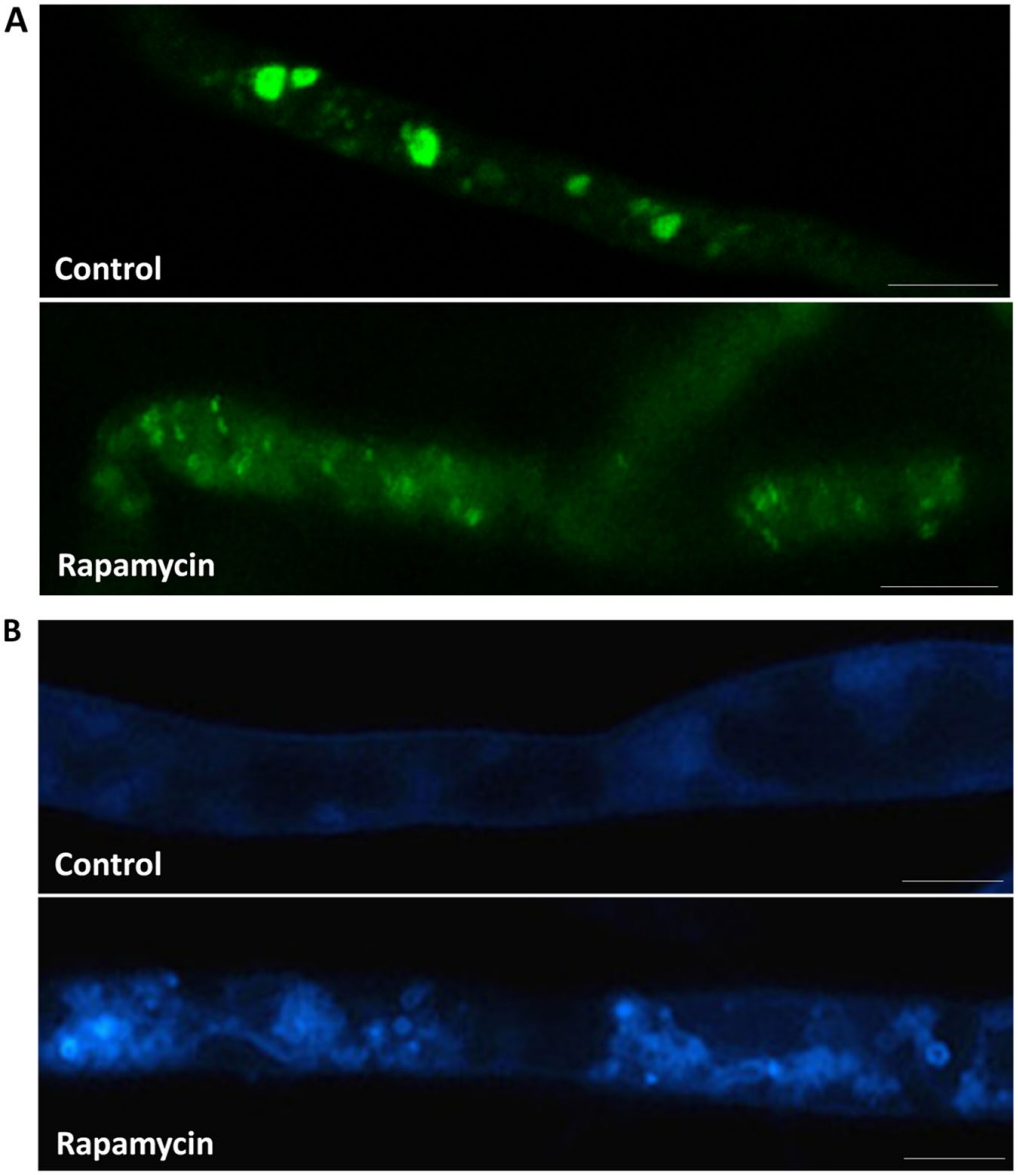
Induction of autophagy in response to rapamycin treatment in *P*. *sojae*. (**A**) Autophagy indicated by GFP-PsAtg8. GFP-PsAtg8-expressing *P. sojae* hyphae were incubated in 10% V8 juice. After treated with 100 nM rapamycin or DMSO (control) for 4 h, hyphae were analyzed by confocal microscopy. Bars = 10 μm. (**B**) Autophagy indicated by MDC dye. *P. sojae* wild-type hyphae were incubated in 10% V8 juice. After treated with 100 nM rapamycin or DMSO (control) for 4 h, hyphae samples concurrently stained with MDC were analyzed by confocal microscopy. Bars = 10 μm.

Rapamycin, a lipophilic macrolide antibiotic, is a well-established autophagy inducer^35^. Here, we used three different assays to test rapamycin-induced *P. sojae* autophagy. The first assay measured the altered location of a GFP-Atg8 fusion protein after processing. During autophagy, Atg8 is normally incorporated into the autophagosome, delivered into the vacuolar lumen, and degraded by vacuolar hydrolases. When the fusion protein undergoes the same process, the stable GFP protein is released^36^. Thus, Atg8 is the most common marker used to monitor autophagy. In *P. sojae*, one highly conserved homolog to Atg8 (PsAtg8) was identified (Figs 1 and 3A), and a plasmid with a *GFP*-*PsAtg8* fusion gene driven by the constitutive *Ham34* promoter was used for transformation. *GFP*-*PsAtg8*-overexpressing transgenic lines were generated using the polyethylene glycol (PEG)-mediated protoplast stable transformation method in *P*. *sojae* ^37^. Using fluorescence microscopy, we found that GFP-PsAtg8 is targeted to the PAS in control (treated with DMSO) mycelia and appeared as elliptical spots. When mycelia were treated with 100 nM rapamycin for 4 h, a decrease in the GFP-PsAtg8 signal was clearly observed (Fig. 5A). Some GFP-PsAtg8 puncta persisted within the vacuole lumen, whereas the majority of PsAtg8 molecules were degradated, and GFP molecules were released into the cytoplasm (Fig. 5A).

The second assay examined autophagosomes stained with monodansylcadaverine (MDC), a dye that has been widely used as an indicator of autophagic activity^38^. As shown in Fig. 5B, control *P. sojae* mycelia exhibit hardly any MDC fluorescence. After mycelia were incubated in 100 nM rapamycin for 4 h, the punctate fluorescent dots from MDC-stained autophagosomes substantially increased in number. It was also found by electron microscopy that more autophagosomes accumulated in the cytoplasm in rapamycin treatment hyphal cells (Supplementary Fig. S2A and S2B). These observations indicated that autophagy was induced in *P. sojae* by rapamycin as in other eukaryotes.

### Autophagic activation in *P. sojae* sporangia and germinating cysts

**Figure 6:**
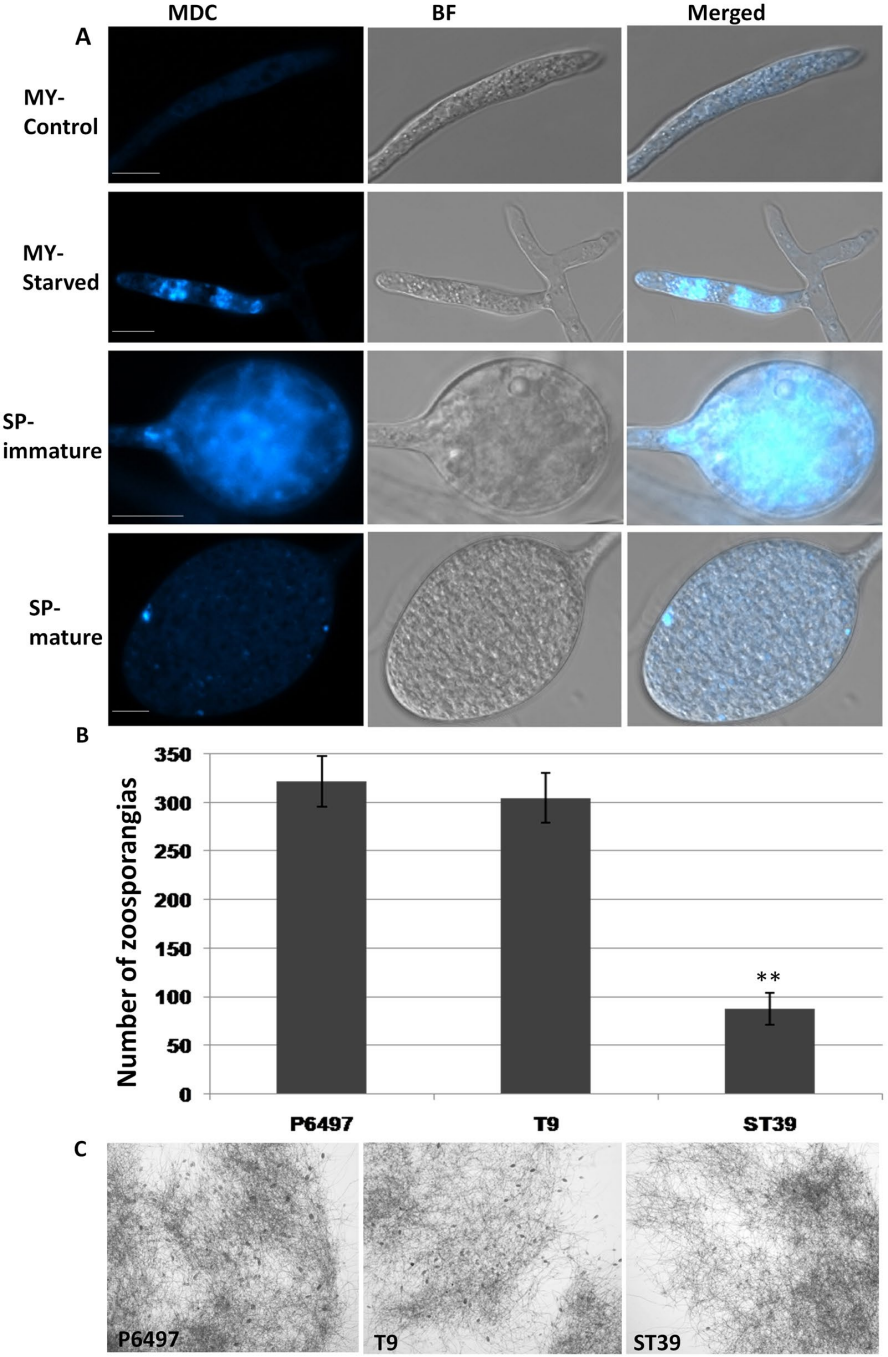
*P. sojae* sporangial production is regulated by autophagy. (**A**) Visualization of autophagic activation with MDC in *P*. *soaje* mycelia (MY) and zoosporangia (SP). Wild-type hyphae were incubated in 10% V8 juice for 30 h. After 3 washes and incubation with sterile distilled water for 4 h, hyphae were concurrently stained for MDC and analyzed by confocal microscopy. Bars = 10 μm. (**B**) Numbers of sporangia produced by *PsAtg6a* transgenic lines. Sporangia of the indicated samples were counted 12 h after the induction of sporangial production. Representative data are shown from three separate experiments. The bars indicate standard errors, and stars above bars indicate that the difference from the wild-type value was significant. **P < 0.01 (*t*-test). (**C**) Sporangial phenotypes for*PsAtg6a* transgenic lines. Micrographs were taken 12 h after sporangial induction. Black arrows indicate sporangia. Bars = 500 μm.

Sporangia, part of the asexual life cycle of *Phytophthora* species, play key roles in diffusion and infection^39^. In *P*. *sojae*, sporangia are induced under nitrogen starvation conditions, which a method used to induce cell autophagy in fungi such as *S. cerevisiae* and various filamentous fungi^4^. The dependence on starvation indicates the possible involvement of autophagy in sporulation. To test whether autophagy is activated during sporulation in *P. sojae*, sporangia were collected and stained with MDC. As shown in Fig. 6A, MDC fluorescence was hardly detected in mycelia cultured in 10% V8 juice. When fresh hyphae were washed with sterile distilled water 3 times and incubated in sterile distilled water for 4 h for sporulation, punctate fluorescent MDC-stained autophagosomal dots increased robustly in some hypha apexes. Furthermore, strong MDC fluorescence was observed in immature sporangia, but very little staining was observed in mature sporangia (Fig. 6A). In addition, when the *PsAtg6a* gene was silenced in *P. sojae*, substantially reduced sporulation was detected (Fig. 6B,C). These results suggest that autophagy might play an important role in sporulation and that it is specifically activated during the proliferation of sporangia from the expansion of hypha apex to the subdivision of cytoplasm in sporangia but during not the release stage.

**Figure 7:**
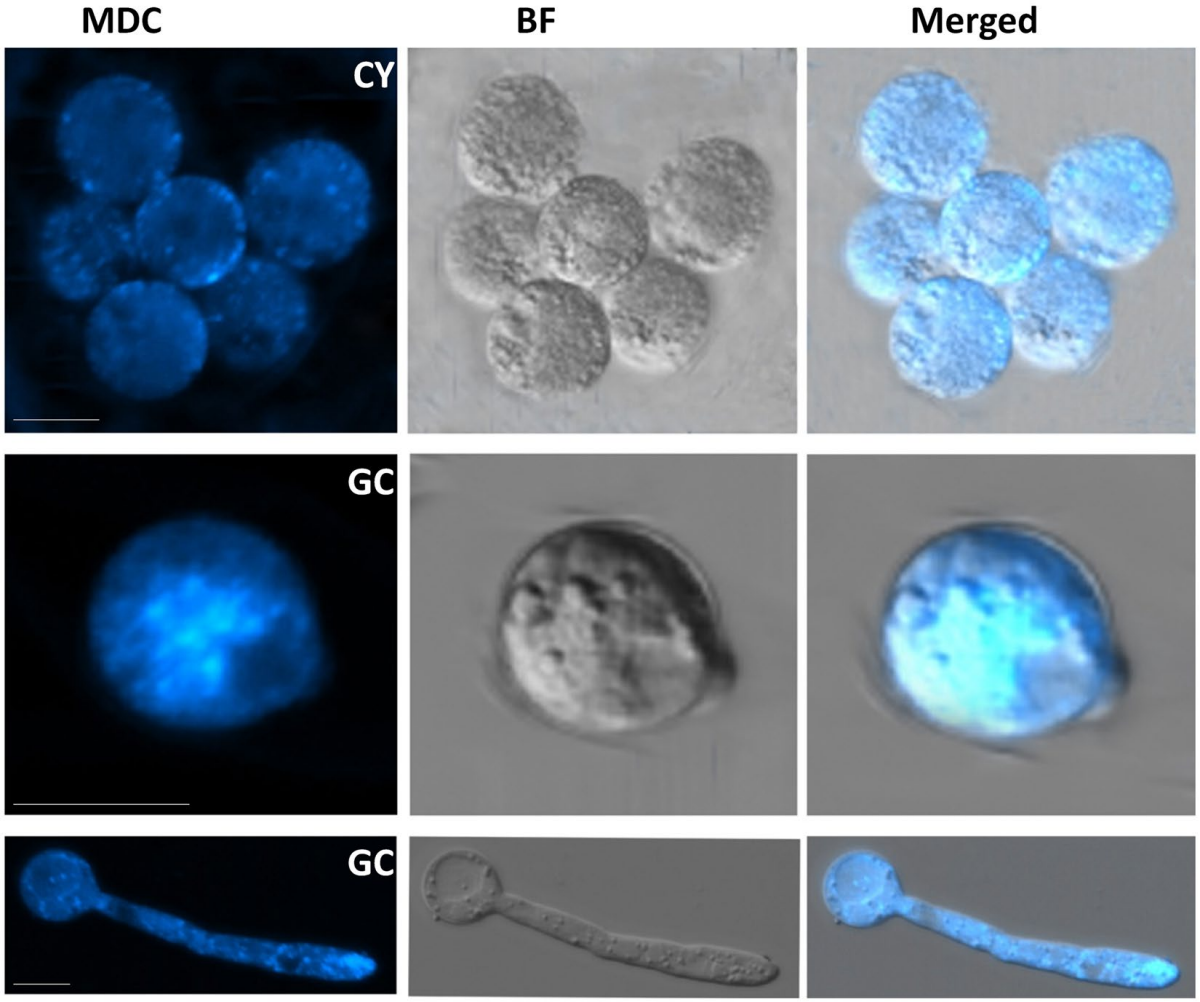
Autophagosome formation in the germinating cyst stage of *P. sojae*. Visualization of autophagy activation with MDC in cysts (CY) and germinated cysts (GC) of *P*. *sojae*. Cyst and germinated cyst samples were collected, concurrently stained with MDC, and analyzed by confocal microscopy. Bars = 10 μm.

Next, more tissues, including cysts and germinating cysts, were stained with MDC. Zoospores are generally short-lived and quickly differentiate to form cysts, so fluorescence images of swimming zoospores were not detected. As shown in Fig. 7, little staining was detected in resting cysts. However, MDC fluorescence remarkably increased when cysts started to germinate, and there was a slight reduction in fluorescence as the germ tube grew. This pattern of autophagosome formation indicated that the autophagy process was activated during cyst germination, making it plausible that autophagy plays a role in infection.

### Construction of *PsAtg6a* transgenic lines

**Figure 8:**
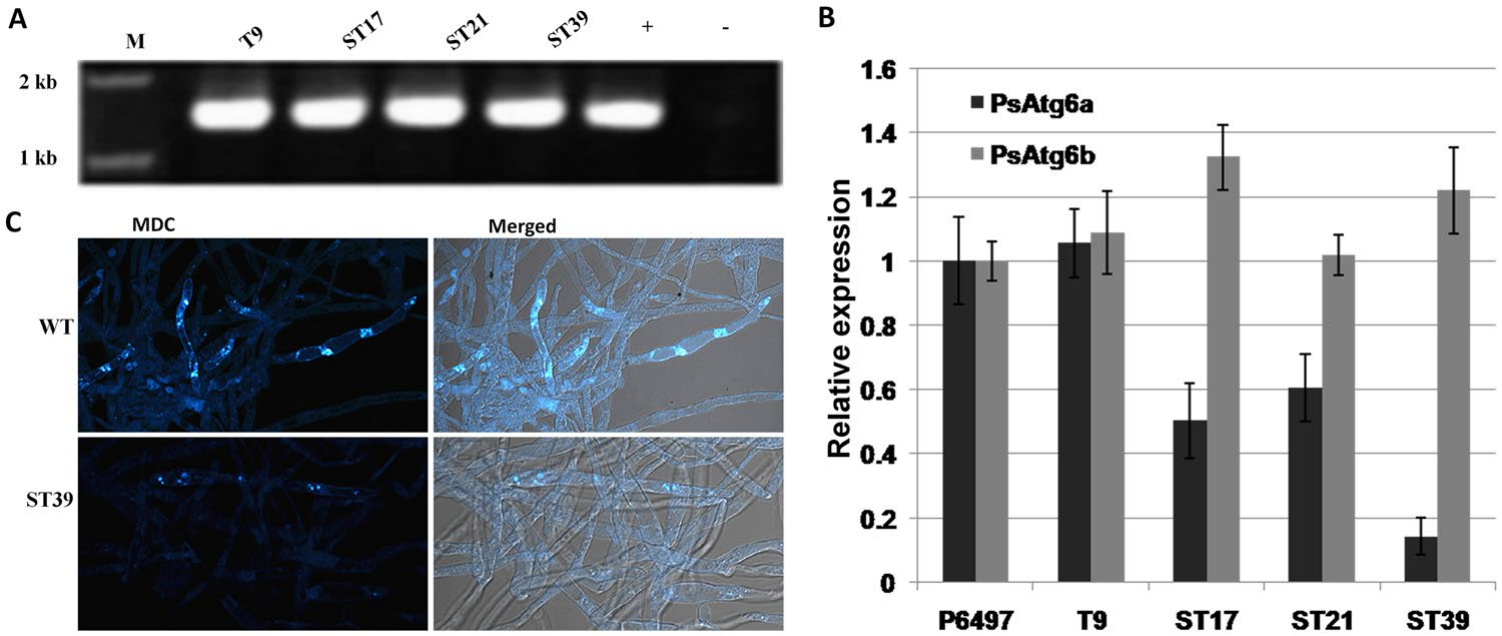
Construction of *PsAtg6a*-silenced lines. (**A**) Verification of incorporation into genomic DNA by PCR using oligonucleotides from the Ham34 promoter and terminator regions as primers. The transgene should yield an amplified fragment 1520 bp in length. +: plasmid DNA; WT: wild-type strain P6497; M: molecular markers. (**B**) qRT-PCR measurement of the relative transcript levels of *PsAtg6a* and *PsAtg6b* in control (P6497), control (T9) and silenced (ST17, ST21 and ST39) transformants. The relative expression levels were calculated using *TEF1* as the reference gene. The bars indicate standard errors, and stars above the bars indicate that the difference from the wild-type value was significant. (**C**) Visualization of autophagic activation with MDC in wild type and ST39. *P. sojae* wild-type or ST39 hyphae were incubated in 10% V8 juice for 30 h. After 3 washes and incubation with sterile distilled water for 4 h, hyphae samples were concurrently stained with MDC and analyzed by confocal microscopy. Bars = 10 μm.

To further investigate the role of autophagy in the sporulation and pathogenicity of *P*. *sojae*, *PsAtg6a*-silenced transgenic lines were generated using the polyethylene glycol (PEG)-mediated protoplast stable transformation method in *P*. *sojae* ^37^. Atg6, the first identified marker protein for autophagy, is part of a lipid kinase complex and plays a central role coordinating the cytoprotective function of autophagy. In many cases, Atg6 is examined to identify the role of autophagy^40^. In *P*. *sojae*, two Atg6 homologs, PsAtg6a (Ps128490) and PsAtg6b (Ps136998), were identified (Fig. 1). Predictions of the conserved domains showed three conserved β-sheet-α-helix repeats in the autophagy-specific BARA domain (Supplementary Fig. S3A). The phylogenetic tree analyses indicated that PsAtg6a forms a close cluster with AtAtg6, whereas PsAtg6b is close to the Atg6 proteins of *H. sapiens* (Supplementary Fig. S3B). Because the transcriptional and qRT-PCR analysis showed that *PsAtg6a* genes were highly up-regulated during infection, *PsAtg6a* was selected for further study (Fig. 4, Supplementary Fig. S1). The required transformants were preliminarily identified using genomic PCR analysis (Fig. 8A), and the expression levels of target genes were measured using qRT-PCR. As shown in Fig. 8B, *PsAtg6a* transcription levels were reduced to 50%, 60% and 14% of the wild-type value in lines ST6–17, ST6-21 and ST6-39, respectively. The *PsAtg6a* gene in T9 was similar to WT, which was used as a control strain. The *PsAtg6b* gene in all of the transgenic lines was similar to WT. The growth rates of *PsAtg6a*-silenced strains were similar to that of the wild-type and control T9 (Supplementary Fig. S4A and S4B). Compared to WT, *PsAtg6a*-silenced strains showed decreased accumulation of autophagic bodies in hyphal cells after culture in sterile distilled water for 4 h (Fig. 8C). These results suggest that the autophagic pathway in *PsAtg6a*-silenced strains was affected and PsAtg6a might be responsible for autophagy in *P. sojae*.

### *PsAtg6a* silencing in *P. sojae* reduces virulence

**Figure 9:**
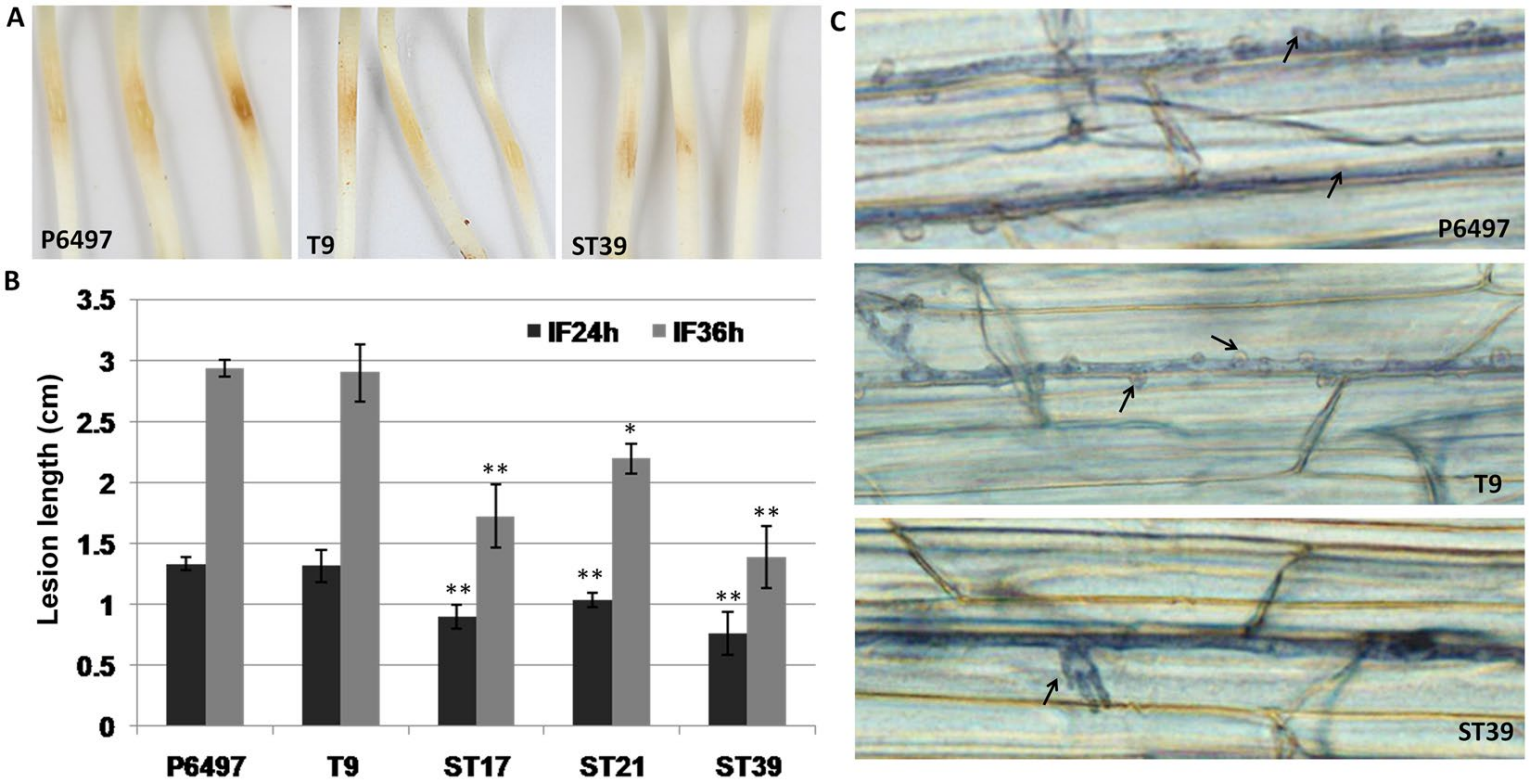
*PsAtg6a* silencing reduced *P*. *sojae* virulence. (**A**) Phenotypes of soybean etiolated seedlings inoculated with wild-type *P*. *sojae* and recombinant strains. Approximately 250 zoospores were used for each inoculation. Photographs were taken 24 and 36 hpi. The edges of the lesions are indicated by arrows. (**B**) Lesion lengths measured at 24 hpi and 36 hpi on five seedlings in each of four independent experiments. The bars indicate the standard errors. **P < 0.01, *P < 0.05 (*t*-test). **(C**) Trypan blue staining of hyphae infecting soybean epidermal cells. Photographs were taken at 30 hpi. Black arrows: haustoria.

To determine whether *PsAtg6a* affected *P*. *sojae* virulence, we inoculated etiolated seedlings of the susceptible soybean cultivar Williams with zoospore suspensions of WT, T9, ST17, ST21, and ST39. Compared to WT and CK (T9), the silenced lines (ST17, ST21 and ST39) exhibited an obvious reduction in virulence against seedlings (Fig. 9A,B). As shown in Fig. 9B, lesions from silenced lines were smaller by approximately 50–80% of WT.

To examine the extent that plant tissue colonization was compromised in *PsAtg6a*-silenced strains, we stained hyphae-infected soybean epidermal cells with trypan blue (Fig. 9C). Many infected hyphae and finger-like haustoria were observed upon infection with WT or CK (T9). In contrast, ST39 hyphae and rare haustoria were observed in the infected soybean epidermal cells. Thus, low expression levels of the *PsAtg6a* gene resulted in a significant reduction in virulence, and the reduced virulence of the *PsAtg6a*-silenced lines seemed attributable to the inability to produce haustoria.

## Discussion

Autophagy, a protein degradation system conserved in eukaryotic cells, is used to recycle macromolecules and aid cellular survival under nutritional starvation conditions^1, 2^. A set of genes involved in this process are called *ATGs* and have been extensively studied in yeast and mammals^19^. In this study, 26 ATGs belonging to 20 different groups in *P. sojae* were identified on the basis of sequence similarity to ATG proteins in yeast and mammals, suggesting that the core components of autophagy are conserved among different species. In *P. sojae*, the compositions of domains within each class were similar with three exceptions (Atg1, Atg11 and Vps34), suggesting possible functional redundancy among different members of the same group. However, the conserved and specific functions of ATGs in the same group still must be further elucidated. In addition, 26 ATGs can be divided into four major functional groups, namely, the Atg1 complex, the phosphoinositide-3-kinase (PI3K) complex, the ubiquitin-like conjugation system and the Atg9 recycling complex.

Over recent decades, autophagy has been shown to play roles in the development and infection process of different pathogenic microbes, including *M. grisea*, *F*. *graminearum* and *U*. *maydis* ^5–9^. However, the roles of autophagy in *P*. *sojae* development and pathogenicity are still unknown. Two autophagy markers^36, 38^, a GFP-PsAtg8 fusion protein and the fluorescent dye MDC, were used to label autophagosomes in *P*. *sojae*. As in other higher eukaryotes, rapamycin and starvation induced autophagy in *P*. *sojae* cells. Furthermore, autophagy was significantly induced during sporangium formation and cysts germination, and *PsAtg6a* silencing suppressed sporulation. These results are consistent with the research on *Phytophthora infestans* showing that autophagy was high in sporangium^41^. Thus, we suggest that autophagy might play an important role in the development of *Phytophthora* species.

According to transcriptome analysis, the relative expression levels of most *ATGs* were significantly increased during infection stages, implying a common role for autophagy in virulence. *PsAtg6a*, a component of the class III PI3K complex, was shown to be essential for pathogenicity. *PsAtg6a*-silenced strains exhibited decreased virulence, and few finger-like haustoria were observed in infected soybean cells. Unfortunately, we were unable to assess autophagy in haustoria using MDC staining because the strong fluorescence signal in the plant cells interfered with observation. In addition, the ST39 line showed no obvious loss of ability to penetrate host cells. It is possible that PsAtg6b might complement a function of PsAtg6a in penetration. Nonetheless, it is clear that autophagy has extensive roles during *P. sojae* development and virulence, and there is a pressing need for further research on the molecular mechanisms underlying autophagy. In addition to these experiments, we attempted to silence other *ATG* genes (*PsAtg1*, *PsAtg6b*, *PsAtg8* and *PsAtg9*) in *P*. *sojae*, but no stable genetic transformants were obtained. CRISPR/Cas9 technology has potential as a tool for *Phytophthora* genetic manipulation, and comparing the roles of autophagy during different developmental and infectious stages should be assessed in future studies.

## Methods

### ATGs identification in oomycetes

To identify ATG proteins in oomycetes, we obtained known ATG proteins from GenBank. Databases for oomycetes were obtained from their original sources: *P*. *sojae* ^42^, *H*. *parasitica* ^43^, *P*. *capsici* ^44^, *P*. *ultimun* ^45^ and *T*. *pseudonana* ^46^. Reciprocal Blastp was used with an e-value cut-off of 1e-5. The retrieved *ATG* genes were used as the query to search oomycete databases with the tBlastN algorithm^47^. Significant hits (E-value < 1e-5) were assessed for the presence of an obvious ATG-related function domain using Pfam^48^. The MUSCLE algorithm was used for multiple protein sequence alignment. The phylogenetic tree of ATG proteins was constructed using MEGA 4.1 with the neighbor-joining method and 1000 replicates were performed for bootstrap analysis^49^.

### RNA isolation and qRT-PCR

Total RNA was extracted from mycelia, sporangia and infected soybean leaves using the RNAsimple Total RNA Kit (Tiangen, China) following the recommended protocol. Quantitative RT-PCR was performed in 20 μL reactions including 20 ng cDNA, 0.2 μM gene-specific primer for the reference *P*. *sojae TEF1* gene (EU079791), 10 μL SYBR Premix ExTaq (Takara), and 6.8 μL ddH_2_O. PCR reactions were performed on an ABI PRISM 7300 fast Real-Time PCR System (Applied Biosystems) under the following conditions: 95 °C for 30 s, 40 cycles at 95 °C for 5 s, and 60 °C for 31 s to calculate cycle threshold values, followed by a dissociation program of 95 °C for 15 s, 60 °C for 1 min, and 95 °C for 15 s to obtain melt curves. The 7300 System Sequence Detection Software (version 1.4; SDS) was used to obtain relative expression levels for each sample. The transcript levels for test genes were determined according to the function ΔC_T_ = C_T_ (test gene) − C_T_ (reference gene). To compare untreated and treated expression levels, the function ΔΔC_T_ was determined using the equation ΔΔC_T_ = ΔC_T_ (treatment) − ΔC_T_ (control), in which the control was mock-treated *P*. *sojae* P6497 mycelia. The induction ratio of treatment/control was calculated using the equation 2^−ΔΔ CT^.

### Rapamycin treatment and monodansylcadaverine (MDC) staining

For rapamycin treatment, hyphae were incubated in V8 for up to 30 h, and 100 nmol/L rapamycin (dissolved in DMSO) was added to 10% V8 medium for 4 h before MDC staining. The same concentration of DMSO was used as a control.

MDC staining was performed as described by Contento *et al*.^50^. Briefly, tissues were stained with 50 μmol/L MDC in PBS for 10 min in the dark, and then washed 3 times with PBS before microscopic observation.

### Plasmid construction and *P. sojae* manipulation

*GFP*-*PsAtg8* is fused with a *AceGFP* gene and the *PsAtg8* gene. To generate pHam*GFP*-*PsAtg8*, the *NptII* gene of pHAMT35N was replaced with the *AcGFP1* gene (AB255038.1) that is amplified using the primers GFP-F and GFP-R. Then, *PsAtg8* was amplified using the primers PsAtg8GFP-F and PsAtg8GFP -R and inserted the 3′ terminal of *AceGFP* gene. To generate pHam*PsAtg6a* for *P. sojae* gene silencing manipulation, the *PsAtg6a* gene was amplified by PCR using primers PsAtg6a-F and PsAtg6a-R. *PsAtg6a* gene was substituted for the *NptII* gene of pHAMT35N^51^.

*P*. *sojae* strain P6497 was routinely grown and maintained on V8 agar. *P. sojae* transformation was performed as previously described^37^. *P*. *sojae* transformants were selected on V8 medium with 50 μg/mL G418 and mycelia were harvested for extraction of DNA or RNA. Mycelial genomic DNA was used to screen for transgenes via PCR using the primers HamF and HamR. Transcription levels were measured using qRT-PCR assay of RNA extracts.

### *P*. *sojae* development and infection assays

*P*. *sojae* development was analyzed using previously described methods^52^. Assays for *P*. *sojae* developmental markers such as vegetative growth, sporangia, zoospores, cysts and cysts germination were performed as previously described^37^. Virulence levels were determined by infecting etiolated soybean (the susceptible cultivar Williams) seedlings with 10 µL zoospore suspension (25 zoospores/µL). The lesion lengths of etiolated seedlings were measured at 24 and 36 hours post infection. Trypan blue staining was performed as described previously^37^. All of the experiments were repeated at least three times, and the data were analyzed using a *t*-test.

### Microscopy

MDC-stained tissues and fluorescent mycelia expressing GFP and GFP-PsAtg8 were examined with the aid of a ZEISS LSM700 Confocal Laser Scanning Microscope (CLSM). ZEN 2010 software was used for fluorescence intensity analysis. An Olympus IX71 instrument was used to count sporangia and to monitor trypan blue staining during infection.

For rapamycin treatment, hyphae were incubated in V8 for up to 30 h, and 100 nmol/L rapamycin (dissolved in DMSO) was added to 10% V8 medium for 4 h. The same concentration of DMSO was used as a control. The mycelial growth was collected, thoroughly washed in distilled water, fixed overnight at 4 °C in modified Karmovsky’s fixative containing 2% paraformaldehyde and 2.5% (vol/vol) glutaraldehyde in 0.1 M phosphate buffer (pH 7.2). The fixed samples were washed three times, for 10 min each time, with 0.1 M phosphate buffer (pH 7.2). The samples were postfixed in 1% OsO4 for 2 h at 25 °C, washed three times with phosphate buffer as before, dehydrated in a graded ethanol series, embedded in Spurr resin, and stained with 2% uranyl acetate and Reynold’s lead solution before sectioning. The ultrathin sections were examined under a JEM-1230 electron microscope (JEOL, Tokyo, Japan) operating at 70 kV. Autophagosomes were counted using Image J software from electron microscopy images of *P*. *sojae* cells in the presence/absence of chemical inhibitors. Autophagosomes were counted using Image J software from electron microscopy images of *P*. *sojae* cells.

## Electronic supplementary material

Supplementary Information
