# Supplementary material for: Network and role analysis of autophagy in Phytophthora sojae
Source: Sci Rep. 2017 May 12;7:1879. doi: 10.1038/s41598-017-01988-7 (PMC5431975; doi:10.1038/s41598-017-01988-7)
Supplement: Supplementary file 1 — Supplementary Information [file 41598_2017_1988_MOESM1_ESM.pdf]

Title:

Network and role analysis of autophagy in *Phytophthora sojae*

Linlin Chen[1], Xiong Zhang[2], Wen Wang[2], Xuejing Geng[1], Yan Shi[1], Risong Na[1], Daolong Dou[2], Honglian Li[1]*

[1] Department of Plant Pathology, Henan Agricultural University, Zhengzhou 450002, China.

[2] Department of Plant Pathology, Nanjing Agricultural University, Nanjing 210095, China.

* Correspondence: Honglian Li, Henan Agricultural University, Zhengzhou 450002, China. Tel: +86-0371-63558791; E-mail: honglianli@sina.com

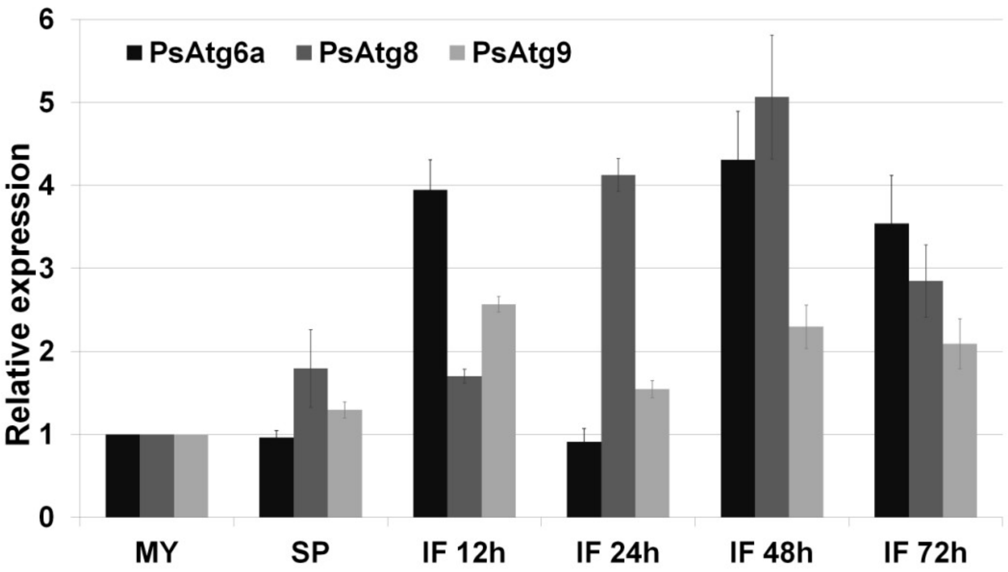

Figure S1. Transcriptional profiles of selected *P. sojae ATGs*. Fold increase in the transcription level fold of the *ATG* genes was calculated relative to the level at MY. The *P. sojae TEF1* gene was used as a reference. Bars represent standard errors from three independent RNA isolations and qRT-PCR replicates.

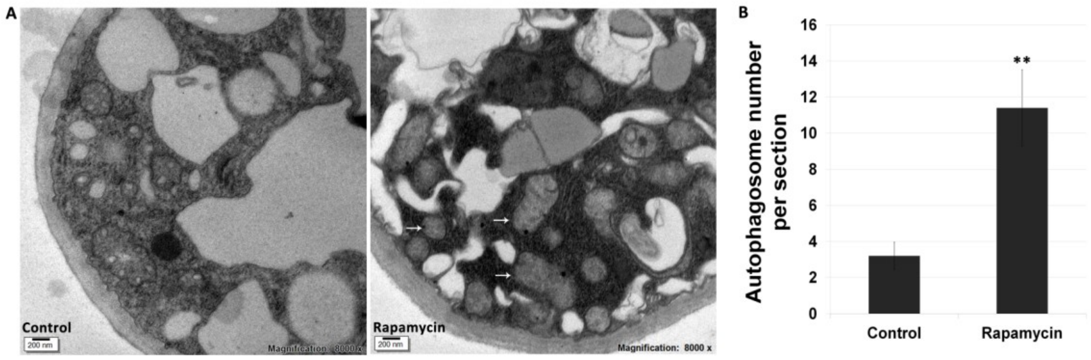

Figure S2. Induction of autophagy in response to rapamycin treatment in *P. sojae*. (A) Transmission electron microscope (TEM) images of *P. sojae* hyphal cells. Autophagosomes (white arrow) accumulate in the cytoplasm in rapamycin treatment hyphal cells (right), but not in the control cells (left). (B) Autophagosomes were counted using using Image J software from electron microscopy images *P. sojae* hyphal cells. $**p < 0.01$.

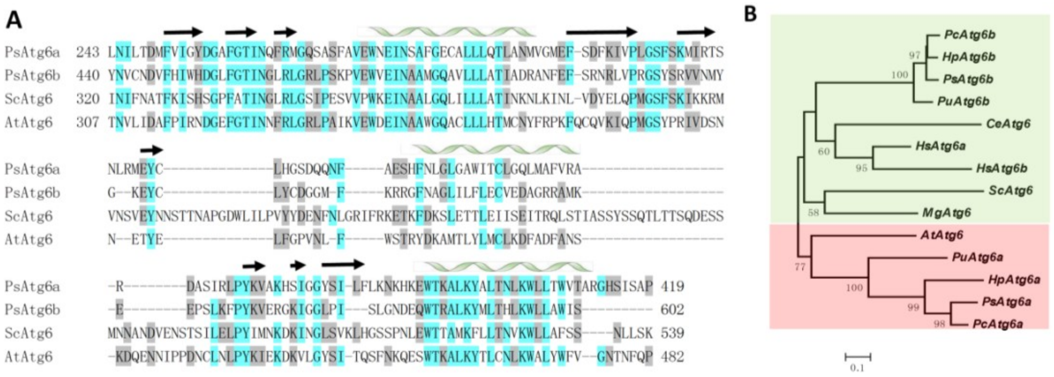

Figure S3. Phylogenetic relationship and sequence alignment of Atg6 proteins. (**A**) The phylogenetic tree includes Atg6 proteins from *P. sojae* (*Ps*), *P. capsici* (*Pc*), *H. parasitica* (*Hp*), *Py. ultimum* (*Pu*), *A. thaliana* (*At*), *C. elegans* (*Ce*), *H. sapiens* (*Hs*), *S. cerevisiae* (*Sc*), and *M. grisea* (*Mg*). Phylogenetic tree was constructed by MEGA 4.1 using the neighbor-joining method with 1000 bootstrap replications. Two clustered branches are displayed in different colors. (**B**) Sequence alignment includes the core conserved C-terminal sequence regions from *P. sojae, S. cerevisiae* and *A. thaliana.* Amino acid sequences were aligned using MUSCLE. The secondary structures of PsAtg6a and PsAtg6b shown above the alignment, were predicted by the Porter algorithm.

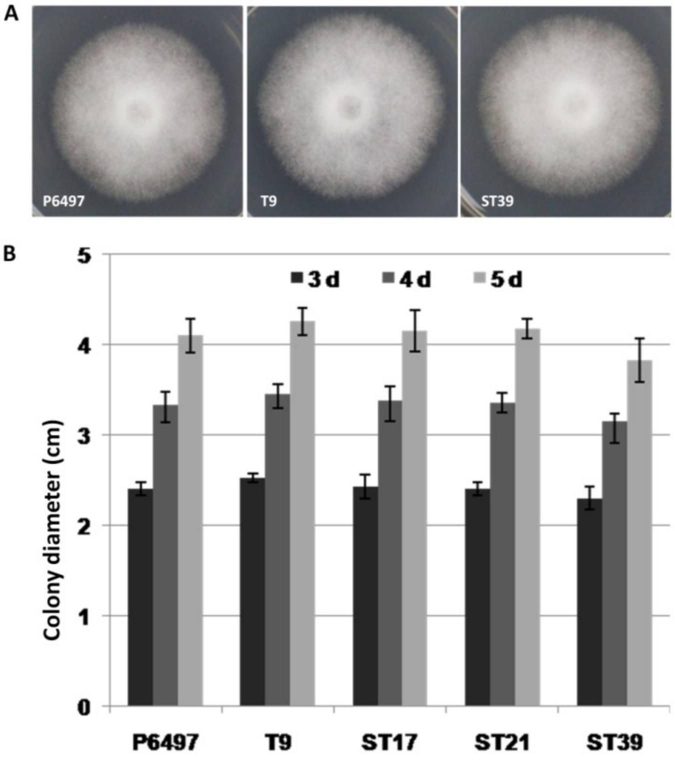

Figure S4. Colony morphology and growth rate of PsAtg6a transgenic lines. None of the transformants showed defects in growth. *P. sojae* colony diameters were measured after culturing on 10% V8 solid medium for 3 days at 25°C in the dark. The data shown are representative of four colonies in each of three independent experiments. Standard errors are marked in brackets.

Table S1. The returned information of each ATG protein from different species.

Cluster	Gene Name	Domain 1	Domain 2	Domain 3
ATG1	*PsAtg1a*	Pkinase	ATG11	ATG11
	PsAtg1b	Pkinase	--	--
	PcAtg1a	Pkinase	--	--
	PcAtg1b	Pkinase	--	--
	PuAtg1a	Pkinase	ATG11	ATG11
	PuAtg1b	Pkinase	--	--
	HpAtg1a	Pkinase	ATG11	--
	HpAtg1b	Pkinase	--	--
	TpAtg1	Pkinase	--	--
	ScAtg1	Pkinase	--	--
	MgAtg1	Pkinase	--	--
	CeAtg1	Pkinase	--	--
	AtAtg1a	Pkinase	--	--
	AtAtg1b	Pkinase	--	--
	AtAtg1c	Pkinase	--	--
	HsAtg1a	Pkinase	--	--
	HsAtg1b	Pkinase	--	--
ATG2	*PsAtg2*	Chorein_N	--	ATG_C
	PcAtg2	--	--	ATG_C
	PuAtg2	Chorein_N	--	ATG_C
	HpAtg2	--	--	ATG_C
	ScAtg2	--	ATG2_CAD	ATG_C
	MgAtg2	--	ATG2_CAD	ATG_C
	CeAtg2	Chorein_N	VPS13_C	ATG_C
	AtAtg2	Chorein_N	ATG2_CAD	ATG_C
	HSAtg2a	Chorein_N	ATG_C	--
	HSAtg2b	Chorein_N	ATG_C	--
ATG3	*PsAtg3*	Autophagy_N	Autophagy_act_C	Autophagy_Cterm
	PcAtg3	Autophagy_N	Autophagy_act_C	Autophagy_Cterm
	PuAtg3	Autophagy_N	Autophagy_act_C	Autophagy_Cterm
	HpAtg3	Autophagy_N	Autophagy_act_C	Autophagy_Cterm
	TpAtg3	Autophagy_N	Autophagy_act_C	Autophagy_Cterm
	ScAtg3	Autophagy_N	Autophagy_act_C	Autophagy_Cterm

	MgAtg3	Autophagy_N	Autophagy_act_C	Autophagy_Cterm
	CeAtg3	Autophagy_N	Autophagy_act_C	Autophagy_Cterm
	AtAtg3	Autophagy_N	Autophagy_act_C	Autophagy_Cterm
	HsAtg3	Autophagy_N	Autophagy_act_C	Autophagy_Cterm
ATG4	*PsAtg4*	Peptidase_C54	--	--
	PcAtg4	Peptidase_C54	--	--
	PuAtg4	Peptidase_C54	--	--
	HpAtg4	Peptidase_C54	--	--
	TpAtg4	Peptidase_C54	--	--
	ScAtg4	Peptidase_C54	--	--
	MgAtg4	Peptidase_C54	--	--
	CeAtg4a	Peptidase_C54	--	--
	CeAtg4b	Peptidase_C54	--	--
	AtAtg4a	Peptidase_C54	--	--
	AtAtg4b	Peptidase_C54	--	--
	HsAtg4a	Peptidase_C54	--	--
	HsAtg4b	Peptidase_C54	--	--
	HsAtg4c	Peptidase_C54	--	--
	HsAtg4d	Peptidase_C54	--	--
ATG5	*PsAtg5*	APG5	--	--
	PcAtg5	APG5	--	--
	PuAtg5	APG5	--	--
	HpAtg5	APG5	--	--
	ScAtg5	APG5	--	--
	MgAtg5	APG5	--	--
	CeAtg5a	APG5	--	--
	CeAtg5b	APG5	--	--
	AtAtg5	APG5	--	--
	HsAtg5	APG5	--	--
ATG6	*PsAtg6a*	--	APG6	--
	PsAtg6b	--	APG6	--
	PcAtg6a	--	APG6	--
	PcAtg6b	--	APG6	--
	PuAtg6a	--	APG6	--
	PuAtg6b	--	APG6	--
	HpAtg6a	--	APG6	--
	HpAtg6b	--	APG6	--
	ScAtg6	--	APG6	--
	MgAtg6	--	APG6	--
	CeAtg6	--	APG6	--
	AtAtg6	--	APG6	--
	HsAtg6a	BH3	APG6	--
	HsAtg6b	--	APG6	--

ATG7	*PsAtg7*	ATG7_N	ThiF	--
	PcAtg7	ATG7_N	ThiF	--
	PuAtg7	ATG7_N	ThiF	--
	HpAtg7	ATG7_N	ThiF	--
	TpAtg7	ATG7_N	ThiF	--
	MgAtg7	ATG7_N	ThiF	--
	ScAtg7	ATG7_N	ThiF	--
	CeAtg7	ATG7_N	ThiF	--
	AtAtg7	ATG7_N	ThiF	--
	HsAtg7	ATG7_N	ThiF	--
ATG8	*PsAtg8*	Atg8	--	--
	PcAtg8	Atg8	--	--
	PuAtg8	Atg8	--	--
	HpAtg8	Atg8	--	--
	TpAtg8	Atg8	--	--
	ScAtg8	Atg8	--	--
	MgAtg8	Atg8	--	--
	CeAtg8a	Atg8	--	--
	CeAtg8b	Atg8	--	--
	CeAtg8c	Atg8	--	--
	AtAtg8a	Atg8	--	--
	AtAtg8b	Atg8	--	--
	AtAtg8c	Atg8	--	--
	AtAtg8d	Atg8	--	--
	AtAtg8e	Atg8	--	--
	AtAtg8f	Atg8	--	--
	AtAtg8g	Atg8	--	--
	AtAtg8h	Atg8	--	--
	AtAtg8i	Atg8	--	--
	HsAtg8a	Atg8	--	--
	HsAtg8b	Atg8	--	--
	HsAtg8c	Atg8	--	--
	HsAtg8d	Atg8	--	--
	HsAtg8e	Atg8	--	--
	HsAtg8f	Atg8	--	--
	HsAtg8g	Atg8	--	--
ATG9	*PsAtg9*	APG9	--	--
	PcAtg9	APG9	--	--
	PuAtg9	APG9	--	--
	HpAtg9	APG9	--	--
	TpAtg9	APG9	--	--
	ScAtg9	APG9	--	--
	MgAtg9	APG9	--	--

	CeAtg9a	APG9	--	--
	CeAtg9b	APG9	--	--
	AtAtg9	APG9	--	--
	HsAtg9a	APG9	--	--
	HsAtg9b	APG9	--	--
ATG10	*PsAtg10*	Autophagy_act_C	--	--
	PcAtg10	Autophagy_act_C	--	--
	PuAtg10	Autophagy_act_C	--	--
	HpAtg10	Autophagy_act_C	--	--
	ScAtg10	Autophagy_act_C	--	--
	MgAtg10	Autophagy_act_C	--	--
	CeAtg10	Autophagy_act_C	--	--
	AtAtg10	Autophagy_act_C	--	--
	HsAtg10	Autophagy_act_C	--	--
ATG11	*PsAtg11*	APG17	ATG11	--
	PcAtg11	APG17	ATG11	--
	PuAtg11	APG17	ATG11	--
	HpAtg11	APG17	ATG11	--
	ScAtg11	--	ATG11	--
	MgAtg11	--	ATG11	--
	CeAtg11	--	ATG11	--
	AtAtg11	APG17	ATG11	--
	HsAtg11	--	ATG11	--
ATG12	*PsAtg12*	APG12	--	--
	PcAtg12	APG12	--	--
	PuAtg12	APG12	--	--
	HpAtg12	APG12	--	--
	TpAtg12	APG12	--	--
	ScAtg12	APG12	--	--
	MgAtg12	APG12	--	--
	CeAtg12	APG12	--	--
	AtAtg12a	APG12	--	--
	AtAtg12b	APG12	--	--
	HsAtg12	APG12	--	--
ATG13	*PsAtg13*	ATG13	--	--
	PcAtg13	ATG13	--	--
	PuAtg13	ATG13	--	--
	HpAtg13	ATG13	--	--
	ScAtg13	ATG13	--	--
	MgAtg13	ATG13	--	--
	AtAtg13a	ATG13	--	--
	AtAtg13b	ATG13	--	--
	HsAtg13	ATG13	--	--

ATG14	*PsAtg14*	ATG14	--	--
	PcAtg14	ATG14	--	--
	PuAtg14	ATG14	--	--
	ScAtg14	ATG14	--	--
	CeAtg14a	Atg14	--	--
	CeAtg14b	Atg14	--	--
	CeAtg14c	Atg14	--	--
	MgAtg14a	Atg14	--	--
	MgAtg14b	Atg14	--	--
	AtAtg14a	Atg14	--	--
	AtAtg14b	Atg14	--	--
	AtAtg14c	Atg14	--	--
	HsAtg14	Atg14	--	--
ATG15	*PsAtg15a*	Lipase_3	--	--
	PsAtg15b	Lipase_3	--	--
	PsAtg15c	Lipase_3	--	--
	PcAtg15a	Lipase_3	--	--
	PcAtg15b	Lipase_3	--	--
	PuAtg15a	Lipase_3	--	--
	PuAtg15b	Lipase_3	--	--
	HpAtg15a	Lipase_3	--	--
	HpAtg15b	Lipase_3	--	--
	HpAtg15c	Lipase_3	--	--
	ScAtg15	Lipase_3	--	--
	MgAtg15	Lipase_3	--	--
ATG16	*PsAtg16*	--	WD40	--
	PuAtg16	--	WD40	--
	HpAtg16	ATG16	WD40	--
	TpAtg16	--	WD40	--
	ScAtg16	ATG16	--	--
	MgAtg16	ATG16	--	--
	CeAtg16a	ATG16	WD40	--
	CeAtg16b	ATG16	WD40	--
	AtAtg16	ATG16	PQQ_2	--
	HsAtg16a	ATG16	WD40	--
	HsAtg16b	ATG16	WD40	--
ATG17	*PsAtg17*	APG17	--	--
	PcAtg17	APG17	--	--
	PuAtg17	APG17	--	--
	HpAtg17	APG17	--	--
	ScAtg17	APG17	--	--
	MgAtg17	APG17	--	--
ATG18	*PsAtg18a*	--	--	--

	PsAtg18b	--	--	--
	PcAtg18a	--	--	--
	PcAtg18b	--	--	--
	PuAtg18a	--	--	--
	PuAtg18b	--	--	--
	HpAtg18a	--	--	--
	HpAtg18b	--	--	--
	HpAtg18c	--	--	--
	TpAtg18	--	--	--
	ScAtg18	--	--	--
	MgAtg18	--	--	--
	CeAtg18a	--	--	--
	CeAtg18b	--	--	--
	AtAtg18a	--	--	--
	AtAtg18b	--	--	--
	AtAtg18c	BCAS3	--	--
	AtAtg18d	BCAS3	--	--
	AtAtg18e	BCAS3	--	--
	AtAtg18f	--	--	--
	AtAtg18g	--	--	--
	AtAtg18h	--	--	--
	HsAtg18a	--	--	--
	HsAtg18b	--	--	--
ATG19	*ScAtg19a*	ATG19_autophagy	--	--
	ScAtg19b	ATG19_autophagy	--	--
ATG20	*ScAtg20*	PX	--	--
	MgAtg20	PX	Vps5	--
	AtAtg20	PX	Vps5	--
ATG21	*ScAtg21*	--	--	--
ATG22	*ScAtg22*	ATG22	--	--
	MgAtg22	ATG22	--	--
ATG23	*ScAtg23*	--	--	--
	MgAtg23	--	--	--
ATG24	*ScAtg24*	PX	--	--
	MgAtg24	PX	--	--
	HsAtg24a	PX	BAR	--
	HsAtg24b	--	--	--
ATG29	*ScAtg29*	--	--	--
	MgAtg29	--	--	--
ATG31	*ScAtg31*	Atg31	--	--
VPS15	*PsVps15*	Pkinase	--	--
	PcVps15	Pkinase	--	--
	PuVps15	Pkinase	--	--

	HpVps15	Pkinase	--	--
	TpVps15	--	--	--
	ScVps15	Pkinase	--	--
	MgVps15	Pkinase	WD40	--
	CeVps15	Pkinase	--	--
	AtVps15	Pkinase	WD40	--
	HsVps15	Pkinase	WD40	--
VPS34	*PsPI3K1*	PI3K_C2	PI3Ka	PI3_PI4_kinase
	PsPI3K2	FYVE	--	PI3_PI4_kinase
	PcVps34a	PI3K_C2	PI3Ka	PI3_PI4_kinase
	PcVps34b	FYVE	--	PI3_PI4_kinase
	PuVps34a	PI3K_C2	PI3Ka	PI3_PI4_kinase
	PuVps34b	PI3K_C2	PI3Ka	PI3_PI4_kinase
	HpVps34b	PI3K_C2	PI3Ka	PI3_PI4_kinase
	HpVps34a	FYVE	--	PI3_PI4_kinase
	TpVps34a	--	--	PI3_PI4_kinase
	TpVps34b	--	PI3Ka	PI3_PI4_kinase
	ScVps34	PI3K_C2	PI3Ka	PI3_PI4_kinase
	MgVps34	PI3K_C2	PI3Ka	PI3_PI4_kinase
	CeVps34	PI3K_C2	PI3Ka	PI3_PI4_kinase
	AtVps34	PI3K_C2	PI3Ka	PI3_PI4_kinase
	HsVps34	PI3K_C2	PI3Ka	PI3_PI4_kinase

Table S2. Oligonucleotides used in the study.

No	Name	Applications	Sequence (from 5' to 3')*
1	GFP-F	Construct of GFP-PsAtg8 fusion gene for *P. sojae* stable transformation	GGGATGGTGAGCAAGGGCGCCGA
2	GFP -R		AAAGGTACCCTTGTACAGCTCATCCATGC
3	PsAtg8GFP-F		AAAGGTACCATGAGCTCATTCAAGAAGGAGCAC
4	PsAtg8GFP -R		AAAGGTACCTTATTGACCGAAGGTGTTTTCGCCGCT
5	PsAtg6a-F	Construct of *PsAtg6a* gene for *P. sojae* stable transformation	GGGacaacaATGTTGCTGCTGCAGAAGCTGCC
6	PsAtg6a-R		AAAGGTACCTTACGAGAAGTGCTCCATGATGACAAC
13	HamF	Screening for putative *P. sojae* transformatns	TTCTCCTTTTCACTCTCACG
14	HamR		AGACACAAAATCTGCAACTTC
15	TEF1F	For quantitative real-time PCR and regular RT-PCR	TGATCGTGCTGAACCACCC
16	TEF1R		CGAGCGACGGTCCATCTT

20	PsAtg6aRTF	For quantitative real-time PCR and regular RT-PCR	TGTCTACACGGTTCGGACCAG
21	PsAtg6aRTR		GACGCGTCTCGAGCCCGAAC
22	PsAtg8RTF		ATTGACAAGAAGAAGTACCTG
23	PsAtg8RTR		TGTTGATGAAGATGAAGATC
24	PsAtg9RTF		AGTCTGTTGGAGGAATCGGTAC
25	PsAtg9RTR		AGGTCGACGATGTGAACGATC
